# Leadless pacemaker implantation in a patient with congenital azygos continuation of the inferior vena cava

**DOI:** 10.1186/s12872-025-04511-3

**Published:** 2025-01-30

**Authors:** Lanyan Guo, Minxia Zhang, Ying Zhao, Qun Yan, Xuyang Feng, Ling Tao

**Affiliations:** Department of Cardiology, Xijing Hospital, Air Force Medical University, Xi’an, Shaanxi China

**Keywords:** Leadless pacemaker implantation, Azygos continuation of the inferior vena cava, Jugular vein

## Abstract

**Abstract:**

In case of venous route abnormality during a leadless pacemaker (LP) implantation, it can be challenging if we still performed via the predesigned femoral vein. We report a patient with normal preoperative laboratory and image results, but azygos continuation of the inferior vena cava (IVC) was suspected during the procedure. Then, we decided to change the implantation strategy, the LP implantation was successfully performed via right jugular vein instead of the classical IVC route. Finally, venous computed tomography (CT) angiography was conducted to testify such venous developmental abnormality.

**Clinical trial number:**

Not applicable

**Supplementary Information:**

The online version contains supplementary material available at 10.1186/s12872-025-04511-3.

## Introduction

Transcatheter leadless pacemaker (LP) has been verified as a pacing alternative to traditional transvenous pacemakers for bradyarrhythmia. Over a 5-year follow-up, the results of the Micra VR (Medtronic Inc., Minneapolis, MN, USA) LP in > 1800 patients highlights the long-term safety and reliability of this novel technology, with low rates of complications and system revisions (both < 5%) [1]. Micra LP was usually implanted via femoral vein in adults, it was not routinely adopted via jugular vein. Herein, we report a case detected with abnormal venous route, and azygos continuation of the inferior vena cava (IVC) was suspected during the LP implantation procedure. Finally, the LP was changed to implant via right jugular vein successfully, and followed by a testify of the venous developmental abnormality via CT angiography.

## Case report

**Fig. 1:**
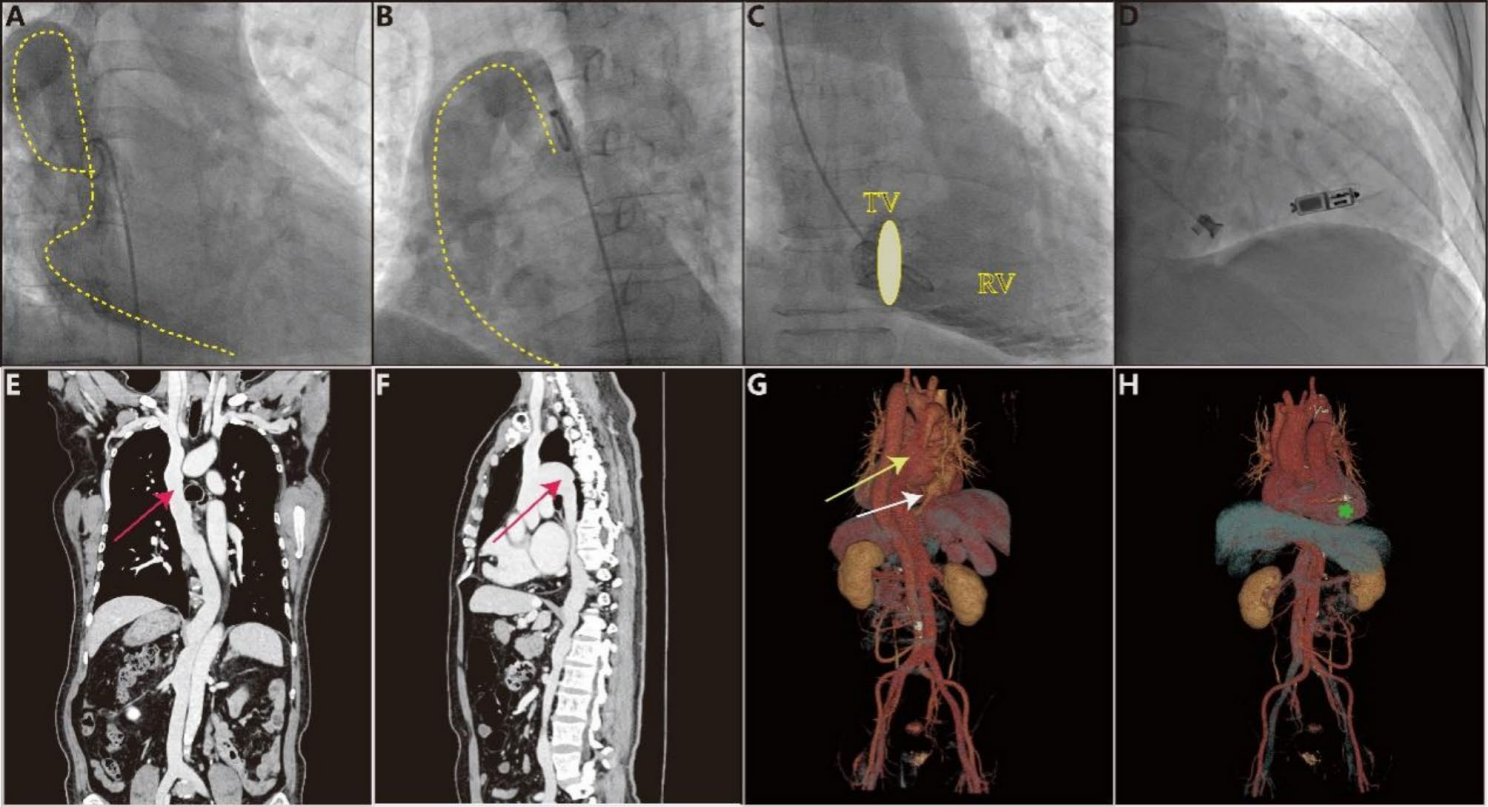
Angiography and CT imaging of the congenital venous anatomy variant, and leadless pacemaker anchored position. **A**, **B**) non-selective venous angiography in RAO and LAO view via femoral vein. The yellow dotted line shows the tortuous and compensatory enlarged azygos vein drainage into RA directly. **C**) non-selective venous angiography of RV via right jugular vein. The yellow oval showed the tricuspid valve (TV). **D**). the leadless pacemaker anchored in RV septal via jugular vein. **E**, **F**) the post-procedural CT imaging of the venous angiography in anteroposterior (AP) and lateral view. The red arrow showed the abnormally tortuous and enlarged azygos. **G**) the 3-D image reconstruction. The yellow arrow showed the abnormally tortuous and enlarged azygos. The white arrow showed the hepatic vein directly drained into RA. **H**) the 3-D image reconstruction in AP view. The green asterisk showed the leadless pacemaker

A 56-year-old male patient with a complaint of intermittent dizziness and amaurosis was referred to a Micra LP implantation. He was diagnosed as sinus arrest, with the longest cardiac asystole of 8.9s. The pre-implant routine examination, including blood tests, transthoracic echocardiography (TTE), coronary computerized tomographic angiography, and chest X-ray, all showed no evident abnormal results. Intra-operatively, when advancing the stiff wire via the right femoral vein, the wire was running in a tortuous route, with a cross to the left side of spinal column and then to the right upper margin of the heart shadow, but failed to reach the inferior vena cava (IVC)/right atrium (RA) (Video S1). Given suspicion of an anomaly of the venous system, non-selective venous angiography was performed, which revealed an enlarged vein draining to the superior vena cava (SVC) (Fig. 1A,B ; Video S2, S3). In consideration of the difficulty and risk during the procedure, the implantation strategy via traditional route was aborted. The right internal jugular vein was punctured utilizing ultrasound guidance, and the wire was easily advanced to the right ventricle (RV). The non-selective RV angiography showed no evident abnormality (Fig. 1C). After successful advancement of the wire via the internal jugular vein, we decided to change our implantation approach. Then an effort was made to slowly and constantly push the delivery system to the RA, and then into RV across tricuspid valve (TV). Finally, the LP implantation was successfully anchored in the right ventricular septal via right jugular vein (Fig. 1D). The pacing parameters at implant were satisfactory, with impedance 1510Ω, threshold 0.38 V (at 0.24ms), and R-wave sensing amplitude 6.6mv. A post-procedural venous computed tomography (CT) angiography was performed, which showed a congenital azygos venous continuation of IVC, then upward draining to SVC and RA, and the hepatic vein drained directly into the RA rather than the IVC. There was no abnormality of SVC course, with a normal reflux to RA (Fig. 1E,H). The patient was discharged without any complications on the second day. The results from 9 months follow up indicated the pacemaker was functioning normally.

## Discussion

The azygos venous system, as a crucial pathway of the posterior thorax, is the most important passage between SVC and IVC. It starts at the junction between the right ascending lumbar vein and right subcostal vein around the T12 Level, then ascends along the right side of the thoracic vertebra, and finally drains into the SVC at the T4-T5 level. Azygos continuation of the IVC is a rare anatomic variant, with an incidence of 0.6%, which is characterized with the tortuous and compensatory enlarged azygos upward draining into RA directly [2]. Usually, patients with this congenital variant are asymptomatic, and unrecognized with routine physical examinations and laboratory tests. However, it brings great challenges for such procedures via IVC, which may change or abort the predesigned operation plan. A similar case has been reported where LP implantation was abandoned and replaced with a staged conventional pacemaker via the SVC [3]. Another report has showed that the LP implantation was performed still through compensatory enlarged and tortuous azygos, which is very difficult and high risky during the procedure [4].

Classical Micra LP was predominantly implanted via femoral vein in adults. While the jugular approach could be an alternative choice, especially for those young children or lack of IVC route [5]. This case indicated that if there is absence of the IVC’s hepatic segments with pre-procedural TTE, a suspicious azygos continuation of the IVC should be considered, and further CT venography should be recommended preoperatively to confirm the anatomy of vascular access. The prompt recognition of such venous abnormality is crucial to determine an appropriate approach before the procedure, which can avoid delays in care and unnecessary expenses. Furthermore, in the condition of congenital azygos continuation of the IVC, we first reported that the LP implantation still can be conducted via alternative jugular vein safely and successfully.

## Conclusion

While rare, congenital azygos continuation of the IVC may complicate the implantation of the LP device. A comprehensive pre-procedural TTE demonstrating absence of the hepatic IVC segment may be a helpful clue suggesting the possibility of this entity, in which case, CT angiography can assist in confirmation. In cases with such anatomical variants, a Micra LP device may still be implanted via an alternative site, such as the jugular vein, successfully.

## Electronic supplementary material

Below is the link to the electronic supplementary material.

**Supplementary Material 1**: **Video 1**: Abnormal IVC route via femoral vein.

**Supplementary Material 2**: **Video 2**: Non-selective venous angiography in RAO view.

**Supplementary Material 3**: **Video 3**: Non-selective venous angiography in LAO view.

## Data Availability

All material and images used in this report are available from the corresponding author by request.

## Abbreviations

AP: Anteroposterior view
CT: Computed tomography
IVC: Inferior vena cava
LAO: Left anterior oblique
LP: Leadless pacemaker
RA: Right atrium
RAO: Right anterior oblique
RV: Right ventricular
SVC: Superior vena cava
TV: Tricuspid valve
TTE: Transthoracic echocardiography

## References

[1] Mikhael F, El-Chami C, Garweg N, Clementy, et al. Leadless pacemakers at 5-year follow-up: the Micra transcatheter pacing system post-approval registry. Eur Heart J. 2024;45(14):1241–51. 38426911 10.1093/eurheartj/ehae101 PMC10998730

[2] Mazzucco A, Bortolotti U, Stellin G, et al. Anomalies of the systemic venous return: a review. J Card Surg. 1990;5:122–33. 2133830 10.1111/j.1540-8191.1990.tb00749.x

[3] Wang X, Ding X, Xu M, et al. Leadless pacemaker implantation and azygos continuation in the inferior vena cava: a case description. Quant Imaging Med Surg. 2023;13:2751–7. 37064385 10.21037/qims-22-885 PMC10102739

[4] Oliveira M, Mesquita D, Cunha PS, et al. Leadless pacemaker implantation via azygos vein in a patient with absence of the hepatic segment of the inferior vena cava. Europace. 2019;21:547. 30698786 10.1093/europace/euy274

[5] Siddeek AE-BH, Hou C, et al. Pediatric Micra leadless pacemaker implantation via internal jugular and femoral veins: experience with 11 patients. Future Cardiol. 2022;18:679–86. 35975839 10.2217/fca-2021-0139

